# The Digital Twin Paradigm in Head and Neck Cancer: Clinical Opportunities and Challenges

**DOI:** 10.2196/95241

**Published:** 2026-11-03

**Authors:** Haolan Yu, Liehao Jiang, Zhuo Tan, Jie Wu, Minghua Ge

**Affiliations:** 1 The Second Clinical Medical College, Zhejiang Chinese Medical University, Hangzhou, Zhejiang, China; 2 Zhejiang Provincial Clinical Research Center for Head & Neck Cancer, Zhejiang Provincial People's Hospital, No. 158 Shangtang Road, Gongshu District, Hangzhou, Zhejiang, 310014, China, 86 0571-85893348; 3 State Key Laboratory for Diagnosis and Treatment of Infectious Diseases, National Clinical Research Centre for Infectious Diseases, Collaborative Innovation Centre for Diagnosis and Treatment of Infectious Diseases, The First Affiliated Hospital, College of Medicine, Zhejiang University, Hangzhou, Zhejiang, China

**Keywords:** adaptive radiotherapy, AI, digital twins, head and neck squamous cell carcinoma, precision oncology

## Abstract

Head and neck squamous cell carcinoma (HNSCC) represents a significant public health challenge. Its treatment is characterized by high anatomical complexity and a critical need for functional preservation of speech, swallowing, and respiration. While multimodality care, including surgery, radiotherapy, and systemic therapy, has substantially improved patient survival, there are still high rates of life-altering toxicities and low response rates to immunotherapy. Current clinical decision-making largely relies on “digital snapshots”—static representations derived from population-based statistics and time point–specific imaging—which usually fail to account for the rapid anatomical and biological change during the course of treatment. Digital twins hold great promise in accelerating scientific breakthroughs and transforming oncology treatment and precision medicine through the creation of high-fidelity virtual patient representations that integrate real-time biological, anatomical, and clinical data. In this viewpoint, we propose 3 interconnected digital twins to establish a conceptual framework for the HNSCC digital twin. These digital twins include the anatomical twin, using virtual surgical planning and augmented reality for geometric precision; the dosimetric twin, using daily imaging and synthetic computed tomography generation for automated adaptive radiotherapy; and the biological twin, integrating deep phenotyping, radiomics, and mechanistic omics to simulate individualized therapeutic trajectories. Beyond acute care, we explore the clinical utility of digital twins in navigating the “de-escalation dilemma” for human papillomavirus–associated disease and improving survivorship through digital phenotyping. With the advent of longitudinal data from medical-grade Internet-of-Things sensors and circulating tumor DNA kinetics, digital twins can serve as active sentinels, detecting functional decline or subclinical events months before clinical manifestation. Furthermore, the implementation of synthetic control arms via virtual populations offers an avenue to accelerate clinical trials and address ethical issues in rare molecular subtypes. Despite this potential, substantial barriers to implementation remain, including profound anatomical instability during treatment, the “black box” nature of deep learning algorithms, and the challenges of multiscale data integration. We provide a technical roadmap for the development of “morphing” digital twins—dynamic systems capable of continuous geometric and biological auditing. By bridging the gap between the macroscopic world of radiation physics and the microscopic world of cellular evolution, digital twins may help shift the management of HNSCC from population-based statistical probability toward more individualized, biologically informed decision-making, with the goal of optimizing HNSCC management while preserving essential patient functions.

## Introduction

Head and neck cancers pose a considerable public health challenge worldwide, with an estimated annual incidence of over 830,000 cases and 430,000 fatalities globally [1]. Head and neck squamous cell carcinoma (HNSCC), the most common histology, constitutes 90% of these malignancies and can develop at many anatomical sites, including the oral cavity, pharynx, and larynx [2]. Treatment of the majority of patients with HNSCC requires multimodality approaches and thus multidisciplinary care, with many patients requiring combinations of surgery, radiotherapy, systemic therapy, and supportive care [2]. While the past few decades have witnessed remarkable advancements in these individual modalities, the integration of their complex data streams remains fragmented. The field is now witnessing a revolutionary transformation driven by digital twins, which hold great promise in accelerating scientific breakthroughs and transforming oncology treatment and precision medicine through the creation of high-fidelity virtual patient representations that integrate real-time biological, anatomical, and clinical data [3].

In the broader medical landscape, digital twin technology has emerged as an important cutting-edge technology to personalize patient care, with many real-world applications already being implemented in cardiology and neurology [4-6]. As demonstrated in cardiovascular health, these digital systems use mechanistic modeling to simulate hemodynamics and predict adverse events like heart failure before they occur [7]. Oncology is now rapidly embracing these technologies to model tumor growth dynamics, drug resistance mechanisms, and radiation dosimetry [8,9]. However, unlike the largely physics-based models in cardiology, digital twins in oncology should account for many additional factors, especially evolutionary biology. These advanced models, achieved through the seamless integration of high-resolution medical imaging, multi-omics data, and clinical data, may offer unprecedented precision in simulating “what-if” treatment scenarios within a virtual framework, shifting the paradigm from population-based statistics to individualized predictive trajectories [10].

In the context of HNSCC, digital twins have the potential to revolutionize care by addressing the intricate balance between maximizing tumor control and minimizing life-altering toxicities. The head and neck region contains a high density of critical structures, including nerves, salivary glands, and swallowing muscles, which are usually located just millimeters from the tumor target [11]. Consequently, treatment necessitates a “multiscale” approach, merging molecular insights (deep phenotyping) with macroscopic anatomical changes (morphological phenotyping), such as the profound weight loss or tumor shrinkage often seen during radiotherapy [12]. The potential of this technology is marked by data fusion, which integrates longitudinal imaging (radiomics), genomic characteristics like human papillomavirus (HPV) status, and continuous patient-reported outcomes tracked through medical Internet of Things (IoT) devices. By serving as the analytical engine for this multimodal data, AI has shown promising performance in predicting complex clinical events, including xerostomia, dysphagia, and locoregional recurrence, although prediction accuracy varies across studies and clinical settings.

For the purposes of this viewpoint, we adopt a spectrum-based definition of the digital twin in oncology, encompassing tools that range from high-fidelity digital snapshots (single time-point patient-specific models) through partial twins (models with limited bidirectional updating) to true digital twins (continuously updated, bidirectional, and simulation-capable systems). Unless otherwise specified, the term “digital twin” refers to this broader spectrum, with the maturity tier of each cited technology noted where relevant.

Although the past decades have witnessed substantial progress in therapeutic measures mainly driven by the divergence in HPV-associated disease and the advent of immunotherapy, there is still a significant gap in how to combine separate data streams into a cohesive decision-support tool for HNSCC. While there have been initial explorations, such as adaptive radiotherapy, these currently represent the lower end of the spectrum, functioning as “digital snapshots” or partial twins, rather than fully continuous, bidirectional digital twin systems. The fundamental shifts from traditional static modeling to a dynamic paradigm across surgical, radiation, and systemic domains are further explained in Table 1. To deconstruct this complexity, this viewpoint proposes a tripartite framework for the HNSCC digital twin. We conceptualize the twin not as a singular entity but as a multilayered framework integrating 3 distinct but interconnected domains: the anatomical twin (geometry), the dosimetric twin (physics), and the biological twin (physiology; Figure 1). This framework builds upon, but is distinct from, prior organizational schemes. Wentzel et al [13] DITTO (digital twin for interventions and temporal treatment outcomes) model emphasizes visual analytics linking interventions to temporal outcomes but does not explicitly stratify domains by data modality or update frequency; a narrative review by Giansanti and Morelli [10] surveys digital twin applications across oncology more broadly without an HNSCC-specific structural decomposition. The tripartite framework proposed here is organized to map directly onto the multidisciplinary decision-making structure of HNSCC care (surgical, radiation oncology, medical oncology, and rehabilitation/speech-language pathology) and is intended to support clinical communication and infrastructure planning rather than to serve solely as a technical taxonomy.

**Table 1.** The paradigm shift from traditional care to digital twins.

Clinical domain	Traditional practice (static modeling)	Digital twin paradigm (dynamic systems)	Primary clinical benefit
Surgical planning	Preoperative VSP^a^ based on single-time-point CT^b^ imaging	Augmented reality overlays and biomechanical tissue modeling	Improved spatial orientation and functional reconstruction prediction
Radiotherapy	Interval-based adaptive RT^c^ using mid-treatment rescans	Continuous dosimetric auditing via daily CBCT^d^-to-synthetic CT pipelines	Reduction in “geographic miss” and decreased toxicity to organs at risk
Systemic therapy	Selection based on TNM stage, p16 status, and population statistics	In silico “what-if” simulations of CRISPR^e^-validated resistance pathways	Sparing of ineffective toxicities (eg, cisplatin) in resistant phenotypes
Survivorship	Reactive surveillance with clinical examinations every 3‐6 months	Proactive monitoring via longitudinal ctDNA^f^ kinetics and IoT^g^ sensors	Earlier detection of subclinical recurrence and functional decline

a VSP: virtual surgical planning.

b CT: computed tomography.

c RT: radiation therapy.

d CBCT: cone-beam computed tomography.

e CRISPR: clustered regularly interspaced short palindromic repeats.

f ctDNA: circulating tumor DNA.

g IoT: Internet of Things.

**Figure 1.**
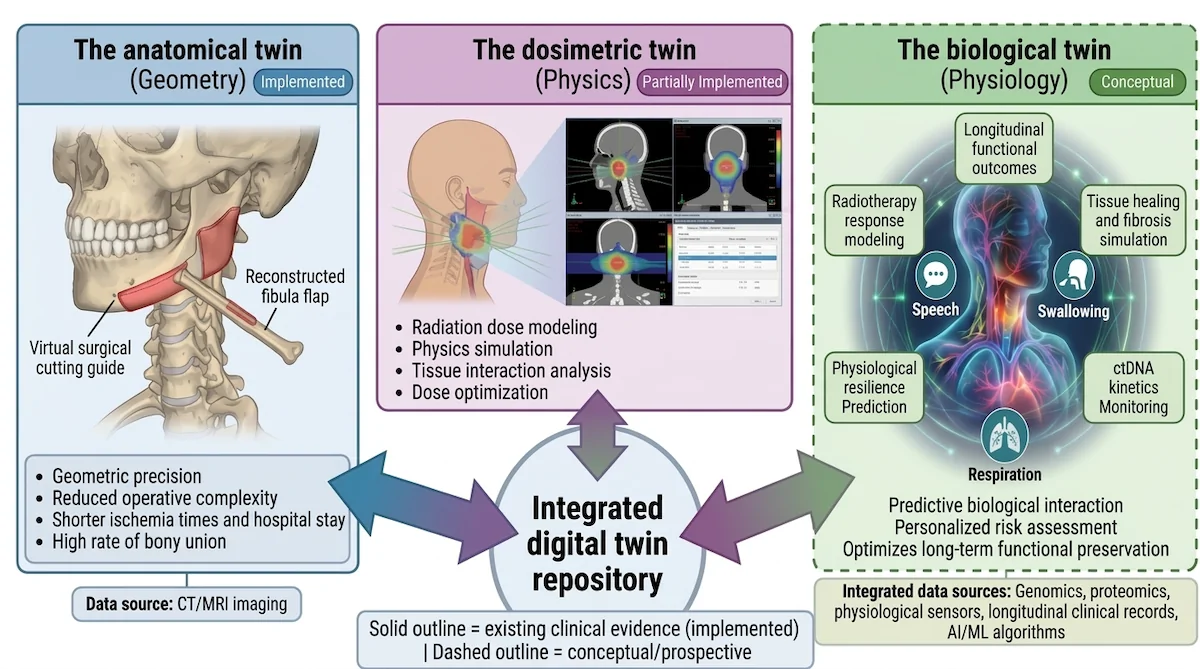
The conceptual framework of the head and neck squamous cell carcinoma digital twin as a multilayered repository. The twin integrates 3 distinct, interconnected dimensions: the anatomical twin (geometry), the dosimetric twin (physics), and the biological twin (physiology), allowing for comprehensive, dynamic patient modeling rather than a sequential process. ctDNA: circulating tumor DNA; CT: computed tomography; ML: machine learning; MRI: magnetic resonance imaging.

## Ethical Considerations

This viewpoint article does not report the analysis of original patient-level data. Because no human participants, patient data, or biological materials were involved, ethical approval and informed consent were not required.

## Elements of Digital Twins in HNSCC: From Static Snapshots to Dynamic Systems

Unlike other solid tumors where monitoring is largely biochemical or radiographic, the management and monitoring of HNSCC require a distinct focus on functional preservation, including speech, swallowing, and respiration. Consequently, a high-fidelity digital twin must act as a “system-of-systems,” covering the following 3 dimensions to capture the patient’s reality.

### The Anatomical Twin (Virtual Surgical Planning and Biomechanics)

The clinical utility of the static anatomical twin in head and neck surgery, largely realized through virtual surgical planning (VSP), including computer-aided design/computer-aided manufacturing, is supported by a growing body of evidence. A large retrospective series from Newcastle demonstrated that shifting from freehand techniques to VSP for free fibula osteocutaneous flaps has significantly reduced operative complexity and the learning curve for trainees [14]. Consistent with findings by Al-Sabahi et al [15] and Sabiq et al [16], the clinical utility of these virtual models is associated with reduced ischemia times, shorter hospital stays, and higher rates of bony union. More importantly, there is no evidence that the lag time required for virtual planning compromises oncological safety, as resection margins remain consistent with standard outcomes. These findings suggest that the anatomical twin can be a valuable tool for geometric precision and operative efficiency, although further prospective validation is warranted.

Although VSP may help to create a preoperative model with high fidelity, there are still many challenges when translating this “anatomical twin” to the soft-tissue environment due to the lack of rigid bony landmarks. In a proof-of-concept case series, Tel et al [17] evaluated the technical feasibility of importing segmented VSP data into augmented reality wearable headsets (Microsoft HoloLens) to overlay holographic representations of critical neurovascular structures onto the operative field during deep cervical space resections. The operating surgeons reported that this visualization approach was perceived as potentially useful for anatomical orientation. These preliminary observations require prospective validation, including controlled comparison with standard surgical navigation techniques, before any clinical benefit can be attributed to this approach [17].

Despite these advances in geometric planning and intraoperative visualization, most of the current models still fail to account for biological dynamism. For instance, the Newcastle series reported that despite accurate bony reconstruction, late complications such as plate extrusion still affected 14% of cases, often compounded by radiation-induced thinning of the soft tissue envelope [14]. Furthermore, patients receiving palliative care exhibited significantly higher flap failure rates, suggesting that geometry alone cannot predict physiological resilience. This highlights the critical gap in current technology. While VSP ensures the bone fits, it lacks the “deep phenotyping” required to predict how that reconstruction will interact with radiation-induced fibrosis or poor vascular status over time. To bridge this gap, the next generation of anatomical twins should integrate functional outcomes—such as speech and swallowing metrics—and biomechanical modeling to predict long-term tissue viability, rather than just immediate surgical success.

### The Dosimetric Twin (Adaptive Radiotherapy)

Patients with head and neck cancer frequently experience rapid anatomical changes, such as tumor shrinkage or catabolic weight loss, during the 6 to 7 weeks of intensity-modulated radiation therapy [13,18]. In standard intensity-modulated radiation therapy, these morphological changes may lead to deviations from the original radiation therapy, which may cause damage to certain critical structures that should not receive high doses. The dosimetric twin aims to address this by acting as a real-time surveillance engine, integrating daily cone-beam computed tomography (CBCT) or magnetic resonance–guided imaging to calculate the “dose of the day.” This capability may help address the risk of incidental dose escalation that occurs even when daily image guidance (image-guided radiation therapy [IGRT]) is used. As demonstrated in a prospective analysis by Schwartz et al [19], anatomical deformations can cause geographic miss of the target volume or unintended dose spillover into surrounding healthy tissue. Their trial revealed that while standard IGRT could maintain target coverage, it failed to prevent increased dose to the oral cavity and larynx due to these anatomical changes. By implementing an adaptive replanning strategy in which all patients received at least one mid-treatment replan, with a subset requiring a second replan based on the supervising physician’s clinical judgment, they achieved a mean dose reduction of 4.1 Gy (9%) to the ipsilateral parotid gland compared to nonadaptive IGRT. A dosimetric twin would automate this process, continuously monitoring the “integral body dose” to identify when a replan is statistically necessary to spare these bystander structures, rather than relying on scheduled interval scans.

Crucially, the dosimetric twin has the potential to complement subjective clinical judgment with quantitative, model-based estimates, although its accuracy remains dependent on data quality and model validation. Current adaptive protocols are often resource-intensive and rely on ad hoc visual inspection. However, Barragán-Montero et al [20] validated a “traffic-light” protocol that serves as a blueprint for the twin’s decision engine. In this study, researchers tracked daily dose accumulation using strict safety limits. If a patient’s metrics crossed a line—like a parotid mean dose over 30 Gy or a drop in target coverage—or if deviations exceeded a 5% “red flag” tolerance, the system alerted the team for verification. Crucially, they found that refining their definitions, such as shrinking margins from 4 to 2 mm, increased sensitivity to 0.96. This allowed them to cut through the noise and identify the specific patients who actually needed a new plan. In doing so, they transformed the dosimetric twin from a simple observer into a proactive triage tool.

### The Biological Twin (Omics and Immunostaging)

Unlike the structural shifts that anatomical and dosimetric twins address, the biological mechanisms of HNSCC resistance are largely ignored by standard staging protocols. Current practice limits us to static snapshots, such as TNM staging or p16 status, which offer little insight into the temporal evolution of tumor physiology. The biological twin offers a way out of this conundrum. By integrating deep phenotyping (eg, genomics) with dynamic radiomics, it may construct a mechanistic simulation tailored to the specific biology of a patient’s disease.

In the context of HNSCC, the need for this dynamic modeling was recently established by Aliotta et al [21], who developed a “radiobiological digital twin” to predict treatment failure. Their model was initiated with multimodality imaging—specifically diffusion-weighted magnetic resonance imaging (DWI-MRI) to map cell density and dual-tracer positron emission tomography (PET; [^18^F] fluorodeoxyglucose for metabolism and [^18^F] fluoromisonidazole [FMISO] for hypoxia). Crucially, they demonstrated that a static pretreatment model was insufficient; however, after the twin was updated with longitudinal tumor volume measurements during the first week of therapy (“dynamic model refinement”), the twin’s prediction error for the final tumor volume dropped significantly (mean absolute error decreased from 23.4% to 12.5%). In this single-institution computational study, the week 1–updated twin predicted locoregional recurrence with an area under the curve (AUC) of 0.83 and was modeled to identify radioresistant phenotypes earlier in the treatment course than conventional clinical assessment. These findings should be regarded as proof-of-concept; the model has not been externally validated, and prospective, multicenter evaluation is required before this approach can inform clinical decision-making.

Furthermore, the biological twin may play an important role in optimizing systemic therapy, particularly in addressing the widespread resistance to platinum-based chemotherapy. To understand the molecular mechanisms behind this resistance, Ludwig et al [22] turned to genome-wide CRISPR (clustered regularly interspaced short palindromic repeats) profiling. Their analysis successfully mapped these mechanisms and zeroed in on the Notch signaling pathway as the main culprit [22]. Their screening of 18 HNSCC cell lines prioritized 207 pivotal genes, revealing that specific alterations in NOTCH1, NCOR1, and SPANXA1 dictate sensitivity to platinum-based agents. By ingesting a patient’s genomic profile, the biological twin can simulate the cellular response to high-dose cisplatin (100 mg/m²). If the twin detects a “Resistant” molecular signature—characterized, for instance, by specific NOTCH1 frameshift alterations or pathway activation—it can predict therapeutic failure with high probability. This could, in principle, help clinicians avoid the severe nephrotoxicity of ineffective cisplatin, pivoting instead to alternative agents or trials targeting the Notch pathway (eg, γ-secretase inhibitors) to align treatment with the tumor’s molecular wiring.

While genomics defines a tumor’s potential, its physical expansion is governed by biomechanics. A truly high-fidelity biological twin must therefore incorporate multiphase poromechanics to model how the tumor interacts with its physical surroundings. Urcun et al [23] demonstrated this by creating a digital twin of tumor spheroids under mechanical confinement. Their “reactive poromechanical model” successfully distinguished between “solid pressure” (stress from the extracellular matrix) and “fluid pressure” (interstitial fluid dynamics), revealing that mechanical confinement alone is a critical driver of necrosis and phenotype switching [23]. In the context of HNSCC, integrating this physics-based layer is crucial for predicting how a growing tumor mass will compress adjacent cranial nerves or collapse the microvasculature, thereby inhibiting drug delivery.

## Digital Twins in HNSCC: Clinical Perspectives

The implementation of digital twin technology represents a transformative framework that bridges the 2 critical phases of the patient journey: the precision optimization of curative-intent therapy and the longitudinal surveillance of survivorship (Figure 2). Having defined the anatomical, dosimetric, and biological architectures, we now examine how these components converge to address the field’s most persistent clinical challenges.

**Figure 2.**
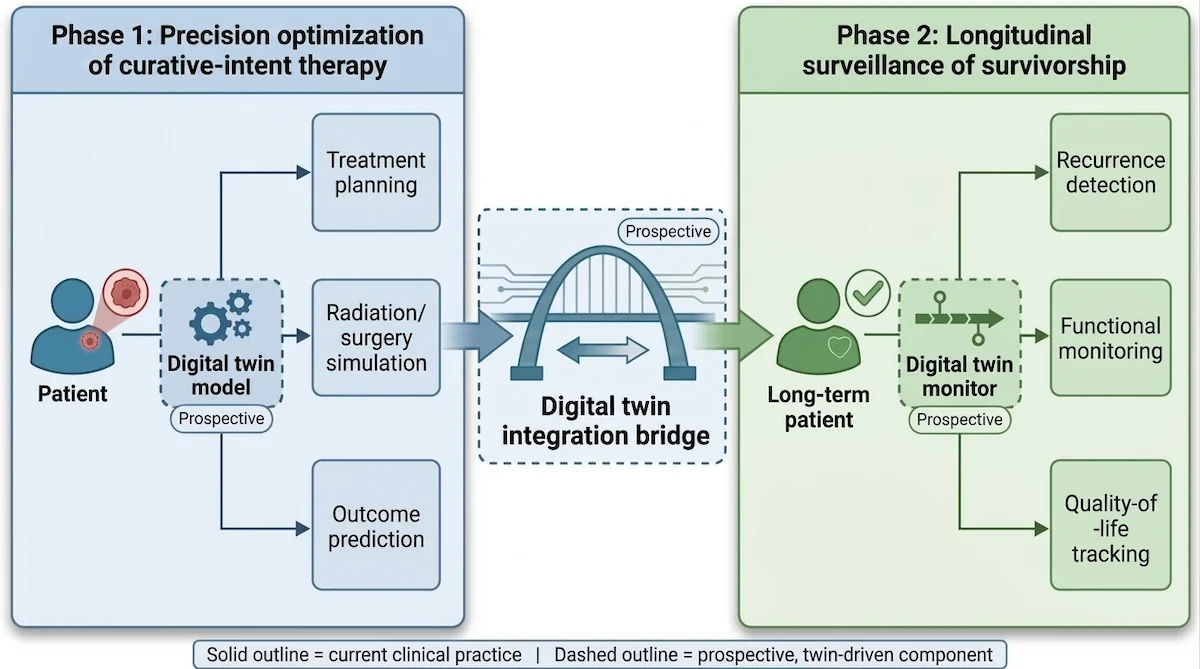
Schematic framework illustrating the role of digital twin technology in bridging curative therapy and long-term survivorship in head and neck oncology. The framework bridges phase 1 (precision optimization of curative-intent therapy: treatment planning, radiation/surgery simulation, and outcome prediction) and phase 2 (longitudinal survivorship surveillance: recurrence detection, functional monitoring, and quality-of-life tracking). Solid outlined elements denote current clinical practice; dashed outlined elements denote prospective, twin-driven components (the digital twin model, the digital twin integration bridge, and the digital twin monitor).

### Optimizing Multimodality Treatment: The De-Escalation Dilemma

One of the most pressing questions in HNSCC is the de-escalation of treatment for HPV-positive oropharyngeal cancer to reduce long-term morbidity without compromising survival. However, the paradigm of using broad clinical criteria (eg, TNM staging) for de-escalation has proven dangerous. Randomized phase III trials, such as De-Escalate [24] and Radiation Therapy Oncology Group 1016 (RTOG 1016) [25], demonstrated that substituting cisplatin with cetuximab resulted in significantly inferior survival and locoregional control, highlighting that clinical “low-risk” features alone are insufficient to guide therapy reduction.

A digital twin offers a risk-free “in silico sandbox” to navigate this dilemma. By generating a virtual patient avatar, clinicians can simulate “virtual clinical trials” for the individual patient. For instance, recent computational studies have used multimodal AI to integrate radiomics with genomic data, successfully identifying the specific subset of patients who are HPV positive and harbor “occult” aggressive traits that lead to treatment failure [26]. A digital twin integrating these predictive algorithms can model whether a specific patient’s tumor biology—characterized by parameters such as hypoxia volume or NOTCH1 mutation status—makes them a safe candidate for reduced-dose radiation (eg, 60 Gy). This shifts the decision from a rigid guideline to a biologically determined prediction, ensuring de-escalation is reserved for patients whose “twin” confirms safety.

Treating HNSCC is rarely a one-time intervention; rather, it is a complex trajectory filled with critical sequential decisions, such as choosing between induction chemotherapy, concurrent chemoradiation, or neck dissection. This is where the “digital twin dyad” becomes essential. It goes beyond simple, static risk scoring to help clinicians navigate these sequential, cascading decisions. Tardini et al [27] tackled this challenge by pairing a “patient twin” (to model biological responses) with a “physician twin” (to simulate treatment protocols) via deep Q-learning. In this retrospective reinforcement-learning simulation, the framework identified, in silico, treatment pathways modeled to improve overall survival by 3.73% and reduce dysphagia rates by 0.75% relative to historical, nonrandomized comparators. However, these estimates are derived from a computational simulation rather than a randomized trial, are subject to the limitations of retrospective historical comparisons, and require prospective validation before they can inform clinical treatment sequencing. The phrase “digital twin dyad” here describes a conceptual modeling construct, not a deployable clinical system.

Translating twin-guided de-escalation into clinical practice will require a prospective, adequately powered randomized trial in which twin-derived risk stratification is prespecified as an enrollment criterion rather than applied retrospectively. Coprimary end points should incorporate both oncologic outcomes (locoregional control and progression-free survival) and functional outcomes (swallowing function and patient-reported quality of life), following the end point structure of De-Escalate and RTOG 1016 to avoid the interpretive inconsistencies that complicated those trials. An independent data safety monitoring board should be charged with detecting interim safety signals arising from twin-prediction errors that lead to inappropriate de-escalation. Finally, early regulatory engagement, for example, a Food and Drug Administration presubmission meeting, is advisable given the current absence of dedicated guidance for trials using algorithmic risk stratification as an enrollment criterion.

### Predicting and Managing Toxicity: The Role of Digital Phenotyping

The head and neck region is an anatomically dense region of critical structures. Late-onset toxicities, such as osteoradionecrosis, severe dysphagia, and fibrosis, can manifest years after therapy. Current surveillance is largely reactive, often identifying functional deficits, such as fibrotic fixation of the swallowing musculature, only after they become irreversible [28].

Digital twins upend the traditional monitoring model by leveraging digital phenotyping, which is a continuous stream of physiological data captured by medical IoT sensors. Recent breakthroughs in wearable technology, for instance, now allow flexible mechano-acoustic sensors to sit on the suprasternal notch, where they monitor swallowing counts and vocal vibrations in real-time [28]. For visual data, algorithms embedded in patients’ smartphones can now map facial kinematics, playing a significant role in measuring jaw movement down to the millimeter [29]. If integrated into a longitudinal digital twin framework, these sensor-derived data streams could, in principle, enable detection of subclinical functional changes, such as reductions in swallowing frequency or alterations in vocal acoustics, earlier in the course of posttreatment functional decline than conventional clinic-based assessment. However, the current studies are feasibility and technical validation reports. These constructs remain theoretical and require prospective evaluation.

### Potential in Clinical Trials: The Synthetic Control Arm

The recruitment of participants for HNSCC clinical trials is notoriously challenging due to the rarity of specific molecular subtypes and the therapeutic toxicity associated with standard therapy. Patients are often hesitant to participate in randomized trials where they risk being assigned to a control arm involving toxic standard regimens (eg, high-dose cisplatin) when promising immunotherapeutic alternatives exist.

Digital twins present a transformative alternative through the creation of synthetic control arms. Unlike static historical controls, modern digital twins use virtual populations, which are statistically weighted cohorts designed to mirror the outcome distributions of actual clinical participants [30]. To achieve high fidelity, researchers are now mining expansive real-world data repositories and applying advanced generative architectures, specifically generative adversarial networks and variational autoencoders, to synthesize these virtual patients [31].

Recent breakthroughs have validated this approach beyond theoretical modeling. Notably, Wu et al [32] used magnetic resonance imaging (MRI)–based digital twins to predict neoadjuvant chemotherapy responses in triple-negative breast cancer, successfully optimizing treatment plans to improve efficacy by more than 20%. By adapting this framework for HNSCC, researchers can use tools such as Madrigal (Zitnik Lab, Harvard Medical School) to generate in silico control arms, which could integrate preclinical datasets to forecast clinical safety outcomes [31], effectively reducing the sample size needed for trials while bypassing the ethical dilemmas associated with control groups. It should be noted that current regulatory engagement with computational modeling, such as that discussed at the 2024 Food and Drug Administration/Medical Device Innovation Consortium symposium [33], has primarily addressed device-specific simulation and defined pharmacological/pharmacokinetic modeling contexts, rather than the broad use of generative AI–derived virtual populations as substitutive control arms in oncology trials. At present, synthetic control arms in oncology are more accurately regarded as a source of supplementary, hypothesis-generating evidence rather than a regulator-accepted substitute for randomized concurrent controls, and their application to HNSCC trials remains exploratory.

### Barriers to Implementation and Technical Roadmap

Although the digital twin promises a paradigm shift in precision oncology, its translation into the clinical reality of HNSCC is obstructed by severe physiological and computational barriers (Figure 3). The head and neck region demands high surgical precision and is characterized by potential anatomical instability, where rapid tumor shrinkage and catabolic weight loss could expose critical structures, such as the parotid glands, to high-dose regions, invalidating the original treatment plan. Alongside this lies the challenge posed by biological heterogeneity. The significant divergence among HPV subtypes and the presence of hypoxic radioresistance complicate generic modeling, particularly when “black box” AI algorithms fail to provide the mechanistic rationale necessary for clinical trust. To bridge these gaps, the technical roadmap must pivot toward “morphing” digital twins and hybrid models. Operationally, a morphing digital twin is defined here as a dynamic system that continuously updates a patient’s 3D anatomical and dosimetric representation through deformable image registration applied to daily CBCT or magnetic resonance–guided imaging; when the accumulated deviation between the virtual patient and the original planned anatomy crosses a predefined clinical threshold, the system automatically flags the case for replanning review—an approach operationalized, for example, by deep learning–based CBCT-to-synthetic-computed tomography (CT) conversion pipelines that enable daily dose recalculation without additional radiation exposure [34]. By fusing deep learning pipelines for daily volumetric adaptation with biologically constrained radiomics, these systems can evolve from static snapshots into continuous geometric auditors. This evolution enables sophisticated strategies like “dose painting” to navigate the disease’s spatiotemporal changes. However, this reality demands a robust infrastructure for multiscale data ingestion and continuous, automated processing (Figure 4). The specific modalities and metrics required to sustain such high-fidelity twins are detailed in Table 2.

**Figure 3.**
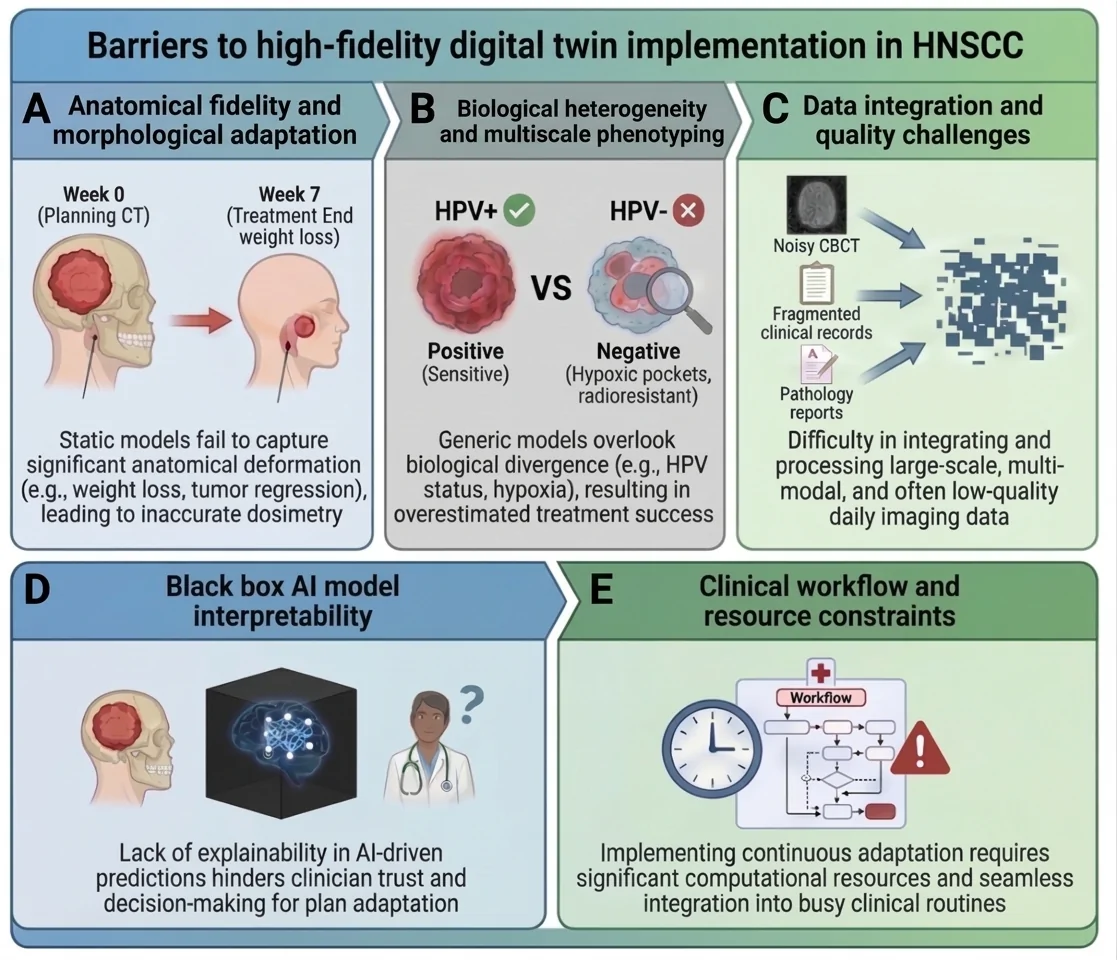
Five key barriers hindering the realization of high-fidelity digital twins in head and neck squamous cell carcinoma (HNSCC), encompassing anatomical, biological, data, AI, and workflow challenges. These barriers include the following: (A) anatomical fidelity and morphological adaptation, (B) biological heterogeneity and multiscale phenotyping, (C) data integration and quality, (D) “black box” model interpretability, and (E) clinical workflow and resource constraints. All panels depict current barriers rather than deployed or proposed technologies. CBCT: cone-beam computed tomography; HPV: human papillomavirus.

**Figure 4.**
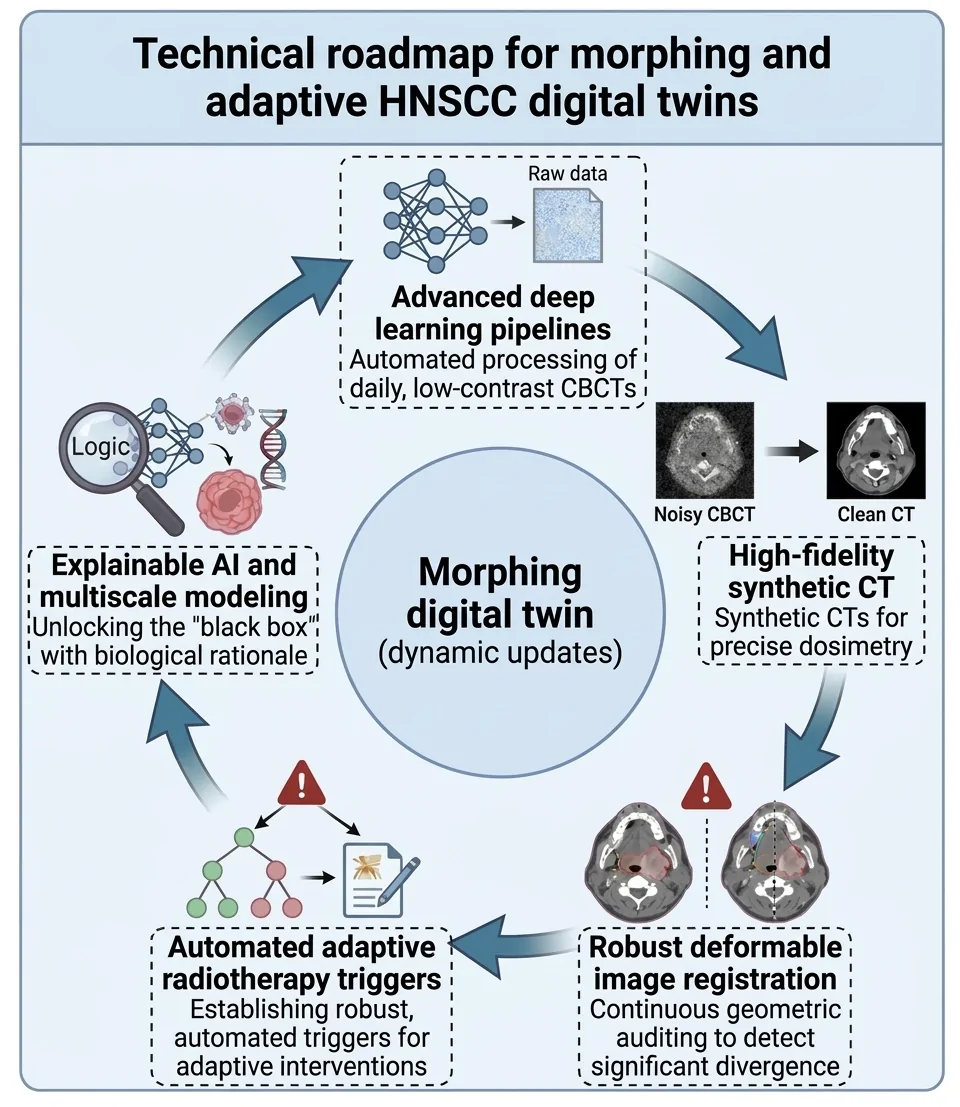
Proposed technical roadmap and future directions for developing morphing, adaptive digital twins in head and neck squamous cell carcinoma (HNSCC), focusing on deep learning, synthetic imaging, registration, automation, and explainability. The roadmap spans advanced deep-learning pipelines, high-fidelity synthetic computed tomography (CT) generation, robust deformable image registration, automated adaptive radiotherapy triggers, and explainable AI. As a forward-looking roadmap, all components are prospective and are shown with dashed outlines; the explainable AI component corresponds to the SHAP (Shapley additive explanations)–based and attention-based methods discussed in the “Biological Heterogeneity and Multiscale Phenotyping” subsection. CBCT: cone-beam computed tomography.

**Table 2.** Multiscale data integration for head and neck squamous cell carcinoma digital twins.

Component	Data modalities (inputs)	Data frequency	Key output metric
Anatomical twin	CT^a^, MRI^b^, AR^c^-projected holograms, IoT^d^ flexible sensors, and smartphone computer vision	Preoperative/intraoperative/continuous (posttreatment)	Geometric precision, margin status, swallowing frequency, and trismus kinematics
Dosimetric twin	Daily CBCT^e^, MR-Linac^f^ imagery, and DVH^g^ parameters	Daily (fractionated)	Cumulative dose accumulation and OAR^h^ sparing
Biological twin	Whole-exome sequencing, CRISPR^i^ profiles, and ctDNA^j^	Interval (baseline/treatment/surveillance)	Therapeutic sensitivity and recurrence probability

a CT: computed tomography.

b MRI: magnetic resonance imaging.

c AR: augmented reality.

d IoT: Internet of Things.

e CBCT: cone-beam computed tomography.

f MR-Linac: magnetic resonance–guided linear accelerator.

g DVH: dose-volume histogram.

h OAR: organ at risk.

i CRISPR: clustered regularly interspaced short palindromic repeats.

j ctDNA: circulating tumor DNA.

### Structural Bottlenecks in Multiscale Data Fusion

Beyond the conceptual challenge of integrating heterogeneous data streams, 4 structural bottlenecks must be explicitly addressed before multiscale digital twins can be realized in clinical practice. First, missing data poses a fundamental problem. Modalities such as circulating tumor DNA (ctDNA) are sampled only at discrete follow-up visits, whereas imaging and IoT data are acquired at markedly different intervals, creating irregular, partially observed time series that challenge dynamic Bayesian updating frameworks. Second, noise and measurement error in low-contrast CBCT images, the primary input for daily dosimetric monitoring, can propagate systematic errors through synthetic CT generation pipelines, directly compromising dose accumulation accuracy. Third, temporal resolution mismatch across data streams, spanning daily fractionated dosimetry, continuous IoT physiological monitoring, and radiological surveillance every 3 to 6 months, represents orders-of-magnitude discrepancies in sampling frequency that cannot be resolved by simple interpolation; multirate alignment approaches, such as hierarchical Bayesian models or multirate Kalman filtering, are required. Fourth, cross-modality consistency is undermined by systematic, vendor-dependent and reconstruction-algorithm–dependent variation in radiomic feature values, which must be corrected through feature harmonization methods, such as ComBat harmonization [30694160], prior to any cross-modal model fusion. Failure to address these bottlenecks at the infrastructure level will limit even the most sophisticated twin architectures to single-institution applicability.

### Anatomical Fidelity and Morphological Adaptation

One of the most immediate barriers to realizing a digital twin for HNSCC is the substantial anatomical instability inherent to the head and neck region. Unlike intracranial malignancies confined within the rigid skull, HNSCC tumors reside in soft tissues that are highly susceptible to weight loss and rapid tumor shrinkage. As demonstrated by Castadot et al [12], the parotid glands often move medially into high-dose radiation regions as the patient loses weight, potentially causing irreversible xerostomia if the treatment plan remains static. Current digital models are largely “snapshots” derived from a single planning CT scan, failing to account for this temporal evolution. Consequently, a static simulation may not be able to predict the accumulated dose to critical structures like the mandible or spinal cord over a 7-week fractionated regimen.

To bridge this gap, future research must focus on the development of “morphing” digital twins capable of dynamic anatomical updates [35]. This requires the integration of deep learning pipelines that can automatically process CBCT images, which are typically low-contrast and noisy, into high-fidelity synthetic CTs for accurate dosimetry, as explored by Çağlar et al [34]. Furthermore, establishing dynamic decision-making for adaptive radiotherapy is essential, as Brouwer et al [36] indicated, the challenge is not only continuous observation but also automation of the decision-making process. Future models are expected to essentially act as continuous geometric auditors, using deformable image registration to alert clinicians when the “virtual patient” diverges significantly from the “planned patient,” thereby necessitating an adaptive intervention.

### Biological Heterogeneity and Multiscale Phenotyping

The predictive power of a digital twin would be substantially diminished if HNSCC were simply treated as a homogeneous disease. This one-size-fits-all approach overlooks the significant biological heterogeneity between HPV-associated tumors and HPV-negative tumors, a difference that directly determines treatment sensitivity. For instance, HPV-negative tumors often contain distinct hypoxic regions, making them a hideout for radiation resistance. As discussed by Spoormans et al [37], theoretical tumor control probability frameworks that neglect these microenvironmental factors are prone to systematic overestimation of therapeutic success. The interpretability deficit of many AI-driven models represents a fundamental barrier to clinical adoption that is distinct from and cannot be resolved by predictive performance alone. A model may achieve high AUC on an internal validation cohort while remaining entirely opaque regarding the biological mechanisms that drive its predictions; such opacity is clinically untenable in high-stakes settings such as HNSCC, where clinicians require a mechanistic rationale before modifying treatment plans. Compounding this, deep learning models are susceptible to performance degradation under distributional shift—that is, when applied to patient populations, imaging equipment, or institutional protocols that differ from the training distribution—a risk that is particularly acute in multicenter deployment. Current explainable AI techniques, including SHAP (Shapley additive explanations) feature attribution and attention visualization maps, offer partial mitigation by identifying which input features most influence a given prediction; however, these methods describe statistical associations rather than causal mechanisms, and their clinical utility in radiation oncology decision-support remains under active investigation.

Consequently, future efforts must embrace “hybrid modeling,” synthesizing the statistical power of radiomics with the physical reality of mechanistic biological constraints. While Rühle et al [38] have successfully demonstrated that radiomics can noninvasively decipher tumor phenotypes, the critical evolution lies in combining these with functional imaging data. By anchoring radiomic features within modalities such as FMISO-PET (fluoromisonidazole–positron emission tomography) or DWI-MRI, we can construct a truly metabolic digital twin. This would enable “dose painting” simulations, where the twin identifies hypoxic cores and simulates the outcome of spatially escalating the radiation dose specifically to those resistant areas. By validating these simulations against spatial transcriptomics, future models could move beyond simple survival prediction to the identification of specific biological pathways, such as hypoxia-inducible factors, that can be targeted with concurrent pharmaceutical agents.

### Functional Prediction and Biomechanical Simulation

In the context of HNSCC, survival is a necessary but insufficient metric of success; the preservation of speech and swallowing function is paramount to quality of life. Current predictive tools, such as the normal tissue complication probability models described by Christianen et al [39], rely on population-level statistics to estimate the risk of dysphagia. While useful for general patient consenting, these statistical models lack the personalized, mechanistic granularity to predict how a specific surgical resection vs radiation fibrosis will alter an individual patient’s muscle kinematics. A surgeon deciding between transoral robotic surgery and chemoradiation currently has no tool to visualize the functional trade-offs of tissue loss vs tissue stiffening for a specific patient.

The future direction lies in the development of “physiome” digital twins that incorporate biomechanical physics engines. Building on computational biomechanical frameworks such as the coupled hard-soft tissue simulation platform described by Stavness et al [40], future research should aim to develop patient-specific models of tongue, hyoid, and pharyngeal actuator dynamics applicable to the posttreatment HNSCC context. Such a physiome digital twin could in principle simulate posttreatment kinematic range of motion and inform pretreatment functional planning; however, the ArtiSynth platform (University of British Columbia) described by Stavness et al [40] is a basic-science bioengineering tool, and its clinical application to individualized HNSCC functional prediction has not yet been demonstrated. Realizing this vision requires substantial prospective development, including patient-specific model personalization, outcome correlation studies, and integration into clinical decision-support workflows.

### Immunological Complexity and the Tumor Microenvironment

While the advent of PD-1 (programmed cell death protein 1) and PD-L1 (programmed death ligand 1) inhibitors has marked significant progress in treating recurrent and metastatic HNSCC, their efficacy is limited to a minority of patients, with objective response rates remaining low, often below 20% [2]. The current clinical biomarker, the combined positive score, is a crude metric that often fails to capture the spatial architecture of the tumor microenvironment. As delineated by Ribas and Wolchok [41], the failure of immunotherapy is often due to physical exclusion, where a dense stroma or “cold” immune environment prevents T-cell infiltration. A digital twin that relies solely on genomic mutational burden without accounting for this spatial exclusion will yield high false-positive predictions for treatment efficacy.

Future research should focus on spatial computational oncology, integrating high-resolution pathology data into the digital twin. Building on the framework proposed by Ribas and Wolchok [41], future models using deep learning approaches applied to histology should map the spatial arrangement of lymphocytes and fibroblasts relative to tumor cells. Specific research directions include simulating the “permeability” of the tumor boundary to cytotoxic T cells and modeling the effects of combination therapies aimed at normalizing blood vessels or disrupting the stromal barrier. By simulating the dynamic confrontation between the immune system and the tumor across spatial dimensions, digital twins may help identify which patients could benefit from immunomodulatory adjuvants prior to checkpoint inhibitor therapy.

### Latency of Surveillance and Liquid Biopsy Integration

The temporal resolution of current oncology models is severely limited by the frequency of physical imaging, which typically occurs every 3 to 6 months. This latency creates a dangerous blind spot in which microscopic recurrence can proliferate undetected. Within the posttreatment scarring characteristic of HNSCC, differentiating between radiation necrosis and tumor recurrence on CT scans is notoriously difficult for radiologists. Reliance on macroscopic imaging results in the digital twin being perpetually “out of date” regarding the patient’s true disease status.

To address this, future digital twins must evolve into dynamic Bayesian systems that ingest high-frequency longitudinal data streams, specifically ctDNA. Chera et al [42] demonstrated that ctDNA kinetics can detect molecular recurrence in HPV-associated oropharyngeal cancer months before clinical manifestation. Research should focus on algorithms that update the digital twin’s recurrence probability in real-time based on blood biomarkers. If the twin detects a rise in ctDNA kinetics while radiological scans remain negative, it could trigger a targeted investigation, such as a localized biopsy or PET scan, guided by probabilistic maps of lymphatic drainage. This shifts the digital twin from a passive documentation tool to an active sentinel for early intervention.

## Conclusions

The management of HNSCC stands at a precipice. While cure rates have improved, the cost of cure, measured in life-altering functional impairment, remains high. Digital twins offer a mechanism to break the “one-size-fits-all” paradigm, shifting from statistical probabilities to more individualized, biologically informed decision-making. By integrating the macroscopic world of anatomy and radiation physics with the microscopic world of genomics and immunology, digital twins have the potential to serve as a valuable decision-support tool, pending further clinical validation. It is essential to recognize, however, that high predictive performance on internal validation datasets does not, by itself, confer clinical trustworthiness; prospective external validation, transparent reporting of model limitations, and robust governance frameworks for AI-assisted decisions are prerequisites for responsible integration. The road ahead requires a concerted interdisciplinary effort to build the infrastructure, prospectively validate the algorithms across institutions, and establish governance frameworks for AI-assisted decisions, with an immediate priority of designing multicenter feasibility trials that test individual twin components against standard of care in head-to-head comparisons.

## Abbreviations

AUC: area under the curve
CBCT: cone-beam computed tomography
CRISPR: clustered regularly interspaced short palindromic repeats
CT: computed tomography
ctDNA: circulating tumor DNA
DITTO: digital twin for interventions and temporal treatment outcomes
DWI-MRI: diffusion-weighted magnetic resonance imaging
FMISO: fluoromisonidazole
FMISO-PET: fluoromisonidazole–positron emission tomography
HNSCC: head and neck squamous cell carcinoma
HPV: human papillomavirus
IGRT: image-guided radiation therapy
IoT: Internet of Things
MRI: magnetic resonance imaging
PD-1: programmed cell death protein 1
PD-L1: programmed death ligand 1
PET: positron emission tomography
RTOG 1016: Radiation Therapy Oncology Group 1016
SHAP: Shapley additive explanations
VSP: virtual surgical planning
